# Fecal Changes Following Introduction of Milk in Infants With Outgrowing Non-IgE Cow's Milk Protein Allergy Are Influenced by Previous Consumption of the Probiotic LGG

**DOI:** 10.3389/fimmu.2019.01819

**Published:** 2019-08-02

**Authors:** Lucía Guadamuro, Maria Diaz, Santiago Jiménez, Cristina Molinos-Norniella, David Pérez-Solis, Juan Miguel Rodríguez, Carlos Bousoño, Miguel Gueimonde, Abelardo Margolles, Susana Delgado, Juan José Díaz

**Affiliations:** ^1^Department of Microbiology and Biochemistry of Dairy Products, Instituto de Productos Lácteos de Asturias (IPLA)-Consejo Superior de Investigaciones Científicas (CSIC), Villaviciosa, Spain; ^2^Instituto de Investigación Sanitaria del Principado de Asturias (ISPA), Oviedo, Spain; ^3^Pediatric Gastroenterology, Hepatology and Nutrition Section, Hospital Universitario Central de Asturias (HUCA), Oviedo, Spain; ^4^Department of Pediatrics, Hospital Universitario de Cabueñes, Gijón, Spain; ^5^Pediatrics Service, Hospital Universitario San Agustín, Avilés, Spain; ^6^Department of Nutrition and Food Science, Universidad Complutense de Madrid (UCM), Madrid, Spain

**Keywords:** cow's milk proteins, non-IgE allergy, fecal microbiota, probiotics, microbial metabolites, protein fermentation, excreted cytokines, introduction of milk

## Abstract

Cow's milk protein allergy (CMPA) is the most common allergy in the first year of life. Non-IgE mediated CMPA is characterized by digestive symptoms and tolerance development before the age of three. Gut microbiota composition in early life has been associated with food allergy. The ingestion of different foods/nutrients may mark different shifts in the microbial colonization of the infant intestine as well as the consumption of probiotics.

**Aim:** To analyze changes in microbiota composition and metabolic and cytokine profiles in fecal samples from infants with non-IgE mediated CMPA after successful milk challenges, tolerance acquisition, and increasing dairy introduction in their diet.

**Methods:** Twelve children with CMPA, aged between 1 and 2 years old, were recruited for the study. Participants were initially consuming hypoallergenic hydrolyzed formulas (four of them supplemented with the probiotic *Lactobacillus rhamnosus* GG), before being exposed to a standardized oral challenge (SOC) with cow's milk. Fecal samples were collected before, 1 week, and 1 month after performing the SOC. Changes in gut microbiota were determined by high-throughput amplicon sequencing of the 16S rRNA gene. Levels of lactobacilli were also determined by quantitative PCR (qPCR). Microbial metabolites were analyzed by chromatographic methods and fecal cytokines related to the Th1/Th2 balance were determined by immunoassay.

**Results:** Lactic acid bacteria significantly increased in infants who outgrew non-IgE CMPA, after the introduction of milk. Microbial metabolites derived from the fermentation of proteins, such as branched chain fatty acids, and *p*-cresol, diminished. After the SOC, some cytokines related to inflammation (TNF-α, IFN-γ) increased. Accompanying the introduction of an unrestricted diet, we found significant differences in fecal microbial composition, metabolites, and cytokines between infants who did not consume the probiotic *L. rhamnosus* GG and those that did.

**Conclusions:** These findings indicate that the introduction of intact milk proteins is followed by modifications in the infant gut environment through changes in immune mediators, microbiota, and its metabolic end-products. Consumption of probiotics during CMPA may contribute to gut homeostasis by fine-tuning these profiles.

## Introduction

Cow's milk protein (CMP) is one of the main causes of food allergy in children, being the primary cause of food allergy in the first year of life (1). Depending on the underlying immunological mechanism involved, CMP allergy (CMPA) can be grouped into three different categories: IgE mediated, non-IgE mediated (NIM-CMPA), and mixed forms (2). While in IgE CMPA, symptoms are generally cutaneous, and respiratory; in NIM-CMPA symptoms are generally digestive. Most of the available tests used for the diagnosis of CMPA, such as skin prick test and the determination of specific IgE to CMP in blood, are based on the determination of IgE. These tests are inadequate for the diagnosis of NIM-CMPA. In this type of CMPA, a clear clinical response after a variable period of an elimination diet, followed by the reappearance of the symptoms with the reintroduction of cow's milk into the infant's diet, are mandatory to obtain a diagnosis (3). Infants with NIM-CMPA usually outgrow their allergy around the second year of life in concurrence with the complete introduction of solid foods and the concomitant maturation of the intestinal microbiota (resembling that of adults) (4). However, the precise molecular interactions leading to tolerance are poorly characterized and not yet fully elucidated. Since no useful laboratory tests to address this allergy resolution are available, repeated oral challenges are usually performed every 6–12 months, in order to evaluate tolerance development, and to avoid continuing a restrictive diet for an unnecessarily long time (5). Current literature in this field indicates that several immunological mechanisms may be responsible for non-IgE reactions. Th1/Th2 imbalances are assumed to have an impact (6).

The importance of the intestinal microbiota, and its effect on health, begins at the early postnatal stage. After birth, the gastrointestinal tract (GIT) of the infant is colonized by different microbial communities that increase in number and diversity reaching a more stable composition at ~2–3 years of age (7). It has been clearly stablished that the type of infant feeding has an influence on the establishment of the gut microbiota (8), and milk, which is a complex food with 3.3% protein content, is a fundamental part of the diet during childhood. However, the impact of the absence of whole milk proteins in the development of the infant's gut ecosystem (in particular in those patients with CMPA following restriction diets) is not well-understood yet. The importance of the correct establishment of gut microbiota in early life and its impact on food allergies has been highlighted (9). In CMPA, only a few studies have focused on studying intestinal microbiota, and its derived metabolites which can drive differential immune development, and food tolerance resolution (10, 11). To date, little information is available on the relationship between tolerance acquisition to CMP and the gut microbiota in NIM-CMPA infants (12), although previous reports described that consumption of some probiotics may facilitate the development of tolerance (13, 14). During infancy the interaction among nutrition, intestinal microbiota, and the immune system is crucial. The aim of the present study was to analyze the changes occurring in feces of infants with NIM-CMPA after successful oral milk challenges, and introduction of conventional dairy products regarding the microbiota composition, microbial metabolites and cytokine profiles.

## Materials and Methods

### Study Design and Infant Patients

Infants aged between 10 and 25 months, and diagnosed with NIM-CMPA, were prospectively recruited for participation at three different regional hospitals in Asturias (Northern Spain): *Hospital Universitario Central de Asturias, Hospital Universitario de Cabueñes* and *Hospital Universitario San Agust*í*n*. All participants had at the inclusion symptoms suggestive of CMPA, a negative skin prick test (wheal diameter < 3 mm), cow's milk-specific IgE values lower than 0.35 kU/l in blood, and a clear positive standardized oral challenge (SOC) performed under medical supervision according to the European Society for Pediatric Gastroenterology, Hepatology, and Nutrition (ESPGHAN) guidelines (15). The study was approved by the Regional Ethics Committee for Clinical Research of the “Principado de Asturias” (Ref. number 105/15). Personal data of the infants providing stool samples conformed to the ethical guidelines outlined in the Declaration of Helsinki and its amendments. Individual informed signed consent was obtained from all the families participating in the study. Exclusion criteria were: use of antibiotics in the previous 3 weeks to stool collection, concomitant presence of other food allergies o other digestive diseases at the inclusion in the study. From this previous cohort of infants (12) we selected those that were consuming different types of hypoallergenic hydrolyzed formulas based on CMP for at least 6 months before a new SOC was performed and who developed tolerance to CMP after the SOC (*n* = 12). For the evaluation of the tolerance development, patients were followed by the corresponding hospital unit; firstly, 1 week after the SOC, by telephone contact with the families who were interviewed for the absence of derived digestive symptoms, and secondly, a month after, in a visit to the clinic where tolerance acquisition, and dairy ingestion were confirmed by the pediatricians involved.

A detailed medical history, including type of feeding, and formula used in the previous 6 months with a restrictive diet, was recorded by the clinicians (Table S1). In this study we inevitably recruited some infants (four of 12) who were consuming the probiotic strain *Lactobacillus rhamnosus* GG (LGG), either as a dietary supplement or included into the composition of the therapeutic hypoallergenic formula during the restriction period. This was mainly due to some previous publications demonstrated that extensively hydrolyzed formula containing LGG, in the market, was able to accelerate immune tolerance acquisition in both IgE and NIM-CMPA (13, 14).

### Collection of Fecal Samples

Families provided three consecutive stool samples from the participating infants: 1 during the exclusion period of intact CMP, 2 or 3 days before performing the SOC (*t* = 0); 2, a week (*t* = 1) after the SOC (during this time the infants consumed at least the recommended daily dose of 200 cc. of milk with intact CMP) and 3, a month after (*t* = 2), when CMP and dairy products were regularly reintroduced into their diet (15). Feces from all participants were collected by their parents in sterile containers after deposition and immediately frozen at −20°C. All samples were thawed on ice once they had been delivered to the laboratory and processed accordingly for different analyses.

### Intestinal Microbial Analysis

#### High-Throughput Amplicon Sequencing and Analysis of 16S rRNA Gene

Fecal samples (0.2 g) were homogenized in 1.8 ml of phosphate buffer saline solution (PBS; pH 7.4) and used for DNA extraction which was performed based on the method of Zoetendal et al. (16) using the QIAamp DNA Stool minikit (Qiagen, Hilden, Germany) with some modifications. The modifications consisted mainly in the addition of an enzymatic lysis step (20 mM Tris-HCl pH 8.0, 2 mM EDTA, 1.2% Triton X-100, and 20 mg/ml lysozyme) before mechanical disruption in a FastPrep FP120 apparatus (Qbiogene, Carlsbad, USA) as previously described (17). The V3 region of the 16S rRNA gene was amplified from the DNA of the samples according to previous reports (18) and 250 bp paired-end sequences were obtained using an Illumina MiSeq System (Illumina, San Diego, USA). The quality-approved, trimmed, and filtered sequences were processed using the Quantitative Insights Into Microbial Ecology (QIIME) software suite and taxonomical classified to the lowest possible taxonomic rank using the SILVA ribosomal RNA gene database v132 as reference, as in previous studies (12). In order to calculate alpha diversity indexes, 16S rDNA sequences were clustered into operational taxonomic units (OTUs), defined at ≥97 % sequence similarity using the UCLUST tool (19). The raw sequences data were deposited in the Sequence Read Archive (SRA) of the NCBI (https://www.ncbi.nlm.nih.gov/sra) under accession numbers SRR6884559, SRR6884560, SRR6884563, SRR6884564, SRR6884570 to SRR6884573, SRR6884575, and SRR6884577 to SRR6884579 for *t* = 0, and SRR7750516 to SRR7750539 for *t* = 1 and *t* = 2.

#### Quantitative PCR (qPCR)

Quantification of the group of lactobacilli in feces was performed by qPCR using the previously described group-specific primers (20) targeting a fragment of 341 bp of the 16S rRNA gene. Amplification reactions were performed in 96-well optical plates (Applied Biosystems Foster City, CA, USA) in two independent experiments using a 7500 Fast Real-Time PCR System (Applied Biosystems). Amplification were done with 2x SYBR Green PCR Master Mix (Applied Biosystems) according to previous reports (17), running samples and no template controls in duplicate wells, and controls for the standard curve in triplicate, into each plate. The standard curve (ranging from 10^2^ to 10^7^ cfu/ml) was calculated using serial 10-fold dilutions of bacterial DNA extracted from a grown culture of *Lactobacillus acidophilus* DSM 20079, the number of cells being calculated by plate counting in MRS (Difco, Detroit, MI, USA) at 37°C. The efficiency was calculated from the slope of the standard curve (*E* = 10^−1/slope^) with Ct values extrapolated by the ABI software (Applied Biosystems). Results were expressed as means of all determinations.

### Analysis of Fecal Metabolites

#### Short-Chain Fatty Acids (SCFAs) Determination

0.1 g weighted of a 1:2 dilution of feces (w/v) in PBS was supplemented with 50 μl of 2-ethyl butyric acid (Sigma-Aldrich, St. Louis, USA) as an internal standard (0.99 mg/ml in methanol) and acidified with 50 μl of 20% formic acid (v/v). The acidic solution was then extracted with 450 μl of methanol and centrifuged for 10 min at 15,700 g. Supernatants were kept at −20°C until analysis in a 6,890 N gas chromatography (GC) apparatus (Agilent Technologies, Santa Clara, USA) connected to a flame ionization detector (FID). All samples were analyzed in duplicate and SCFAs were quantified as previously described (21).

#### Quantifications of Indoles by HPLC

For the determination of 3-methylindole (skatole), *p*-cresol, and indole we used a solid phase extraction method. Preparation of the samples included mixing 0.5 g of fecal samples with 100 μl of the internal standard (2-methylindole from Sigma-Aldrich) at 100 μM, and 2 ml of methanol. The mix was centrifuged (15 min 1,200 g) and supernatants were kept at −20°C for further analysis. Extraction was performed using BondElut-C18 200 mg SPE cartridges (Agilent Technologies) according to previously reported protocol (22) by equilibration and washing steps of the cartridges with TRIS buffer (TRIS 0.05 M, NaCl 0.05 M, pH 8.3). The compounds of interest were finally eluted with 1 ml of acetonitrile and filtered through 0.45 μm PTFE filters (VWR, Barcelona, Spain) before being injected into an Alliance 2695 high-performance liquid chromatography (HPLC) system (Waters, Massachusetts, USA) coupled with a photodiode array (PDA), and fluorescence (FLR) detector modules. The system was equipped with an Alltima® HP C18 250 × 4.6 mm (particle size 5 μm) chromatographic column (VWR) with a precolumn Supelguard Ascentis C18 20 × 4 mm (Supelco, Sigma-Aldrich). The gradient elution was adjusted from that previously described (23) as follows: solvent A contained 0.02 M acetic acid, solvent B consisted of acetonitrile, and solvent C was 2-propanole, applying a linear gradient (min/% A/% B/%C) of: 0/60/30/10; 2.9/60/30/10; 17.8/54/37/9; 18.4/0/100/0. Quantification of indoles was carried out using standard solutions (all from Sigma) for calibration. Data were integrated with the Empower data acquisition software (Waters).

### Cytokine Analysis

The concentration of cytokines related to Th1/Th2 balance was determined using a Bio-Plex 200 system (Bio-Rad, Hercules, USA) and the Bio-Plex Pro Human Cytokine Th1/Th2 Assay (Bio-Rad). Before analysis, feces diluted 10-fold (w/v) in PBS were centrifuged for 15 min at 20,000 g at 4°C, and the supernatants were collected, diluted and treated following the manufacturer's protocol. Standards and samples were determined in duplicate. Data acquisition was performed with the Bio-Plex Manager 6.0 software and the standard curve fitted to a 5 parameter logistic regression.

### Statistical Analysis

Statistical analyses were performed using SPSS v. 25.00 (IBM, Armonk, NY, USA). To examine the differences over time with the ingestion of intact CMPs we used the non-parametric Wilcoxon signed-rank test and two-tailed probability values of *p* ≤ 0.05 were considered significant. The non-parametric Mann–Whitney test was used to compare differences between infants that ingest or not the probiotic LGG. Otherwise specified, medians, and interquartile ranges (Q1 and Q3) were represented in box and whisker graphics using Sigma Plot 14.0 software (Systat software Inc., USA).

## Results

### Changes in the Intestinal Microbiota

The fecal microbiota of a group of 12 infants with NIM-CMPA was analyzed by high-throughput sequencing of 16S rDNA amplicons before (during the exclusion diet), and after successful oral challenges with cow's milk, and further ingestion of dairy products.

After denoising, chimera checking, and trimming reads, we obtained a mean of 58,236 high quality sequences per sample. Taxonomic analysis grouped the sequences mainly into three phyla: Firmicutes, Actinobacteria, and, to a lesser extent, Bacteroidetes.

**Figure 1:**
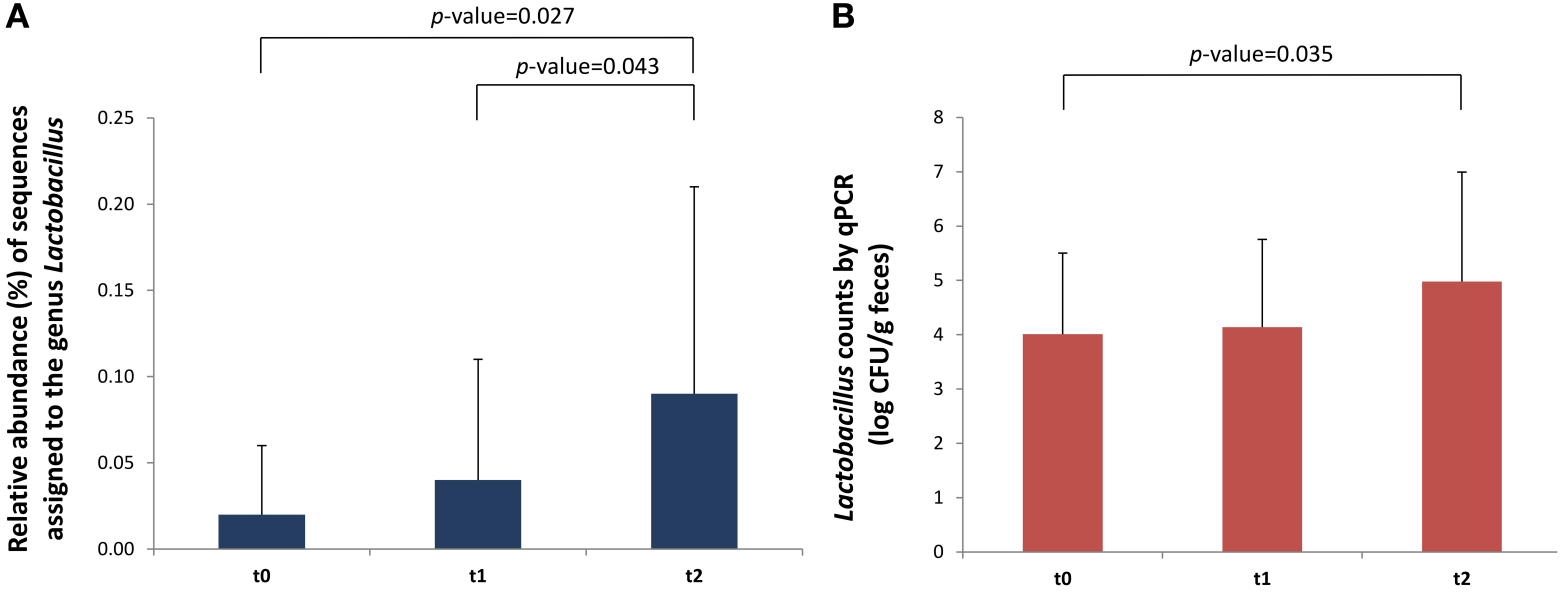
Differences in *Lactobacillus* in fecal samples (*n* = 8) of outgrowing NIM-CMPA infants (excluding those that consumed the probiotic LGG during the milk restriction period). Comparisons were made with the Wilcoxon signed-rank test to examine the differences over time (*t* = 0, *t* = 1, and *t* = 2). **(A)** Relative abundance (%) of sequences belonging to the genus *Lactobacillus* determined through high-throughput sequencing of 16S rDNA amplicons. **(B)** Levels (log cfu/g of feces) of *Lactobacillus* group determined by qPCR. Means and standard deviations are represented.

The relative abundance of several bacterial groups that belong to the *Lachnospiraceae* and *Ruminococcaceae* families significantly decreased 1 month after the introduction of intact proteins and dairy products in the diet (*t* = 2 vs. *t* = 1). These mainly belonged to the group of *Eubacterium hallii* (*Lachnospiraceae*); *p* = 0.038, *Eubacterium coprostanoligenes* (*Ruminococcaceae*); *p* = 0.047, and *Subdoligranulum* (*Ruminococcaceae*); *p* = 0.012 (Table 1). On the contrary, the incorporation of milk to normal diet significantly increased the levels of some fecal lactic acid bacteria (LAB), in particular the genus *Lactococcus* (*p* = 0.018), in comparison with basal time, in which the NIM-CMPA infants were fed hypoallergenic formulas based on hydrolyzed CMP. When analyzing only the group of infants that were not ingesting LGG during the restriction period (*n* = 8), a significant increase in the abundance of the genus *Lactobacillus* over the studied period was also observed (*p* = 0.027). We also noticed increases in the sequences assigned to this genus between a week (*t* = 1) and a month (*t* = 2) after the SOCs (*p* = 0.043) (Figure 1A). These observations were confirmed by qPCR that showed a significant increase, between *t* = 2 and *t* = 0 (*p* = 0.035), in the levels of lactobacilli, again only in the group of NIM-CMPA infants who did not consume LGG (*n* = 8) (Figure 1B). These results are very likely due to the higher counts found at basal time in those infants (*n* = 4) who consumed LGG during the restriction diet (mean of 1.1 × 10^6^ cfu/g) in comparison with those that did not (mean of 1.0 × 10^4^ cfu/g) (Mann–Whitney *U*-test, *p* = 0.016).

**Table 1:** Significant differences in relative abundancies (%) of different bacterial groups in feces of infants outgrowing non-IgE CMPA, before and after ingestion of intact milk proteins with SOC and diet.

**Group/Genera**	***p*-value^a^**	**Relative abundance^b^**		
		***t* = 0 (before SOC)**	***t* = 1 (week after)**	***t* = 2 (month after)**
**ALL INFANTS OF THE STUDY (*****n*** **=** **12)**				
*Eubacterium hallii* group	0.038^#^	0.68 ± 0.56	0.92 ± 0.81	0.56 ± 0.56
*Eubacterium coprostanoligenes* group	0.047^#^	1.39 ± 1.76	1.38 ± 1.27	0.73 ± 0.81
*Subdoligranulum*	0.012^#^	1.64 ± 2.02	2.41 ± 2.38	1.14 ± 1.28
*Lactococcus*	0.018*	0.00 ± 0.00	0.01 ± 0.02	0.11 ± 0.29
**ONLY INFANTS THAT DID NOT CONSUME LGG DURING THE RESTRICTION PERIOD (*****n*** **=** **8)**				
*Lactobacillus*	0.027*	0.02 ± 0.04	0.04 ± 0.07	0.09 ± 0.12
*Eubacterium hallii* group	0.043^#^	0.57 ± 0.57	0.83 ± 0.83	0.47 ± 0.53

a Statistical differences are indicated with an asterisk between t = 2 and t = 0 and with a # between t = 2 and t = 1 (Wilcoxon tests for paired samples).

b Mean relative abundance ± standard deviation.

*CMPA; Cow's milk protein allergy. SOC; standardized oral challenge. LGG; Lactobacillus rhamnosus GG*.

**Figure 2:**
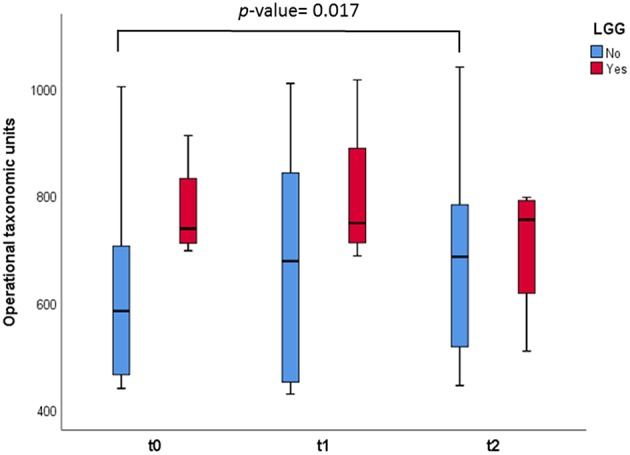
Box and whisker plots representing measurement of OTUs observed in the fecal samples. The infants were divided in two groups: those who consumed the probiotic LGG (*n* = 4, in red), and those who did not (*n* = 8, in green). Comparisons were made with the Wilcoxon signed-rank test to examine the differences over time (*t* = 0, *t* = 1, and *t* = 2).

In relation to alpha diversity we also observed, in samples from those infants who did not consume LGG, significant increases in the number of OTUs when comparing this measure after 1 month of regular dairy consumption with respect to basal time (*p* = 0.017) (Figure 2). Also in this case, the number of OTUs at *t* = 0, with a diet based on hydrolyzed CMP, was numerically higher in NIM-CMPA infants who had consumed probiotic lactobacilli (mean of 770 OTUs) compared to those who did not.

### Changes in Fecal Microbial Metabolites

**Figure 3:**
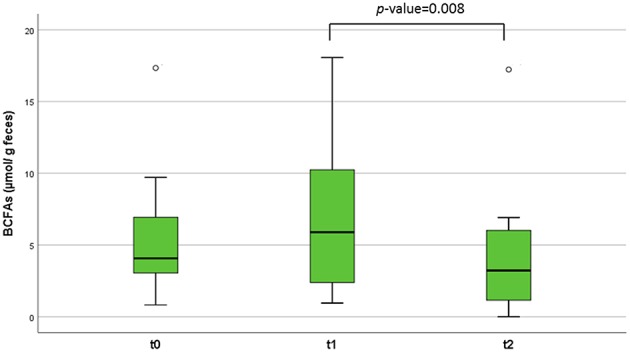
Branched chain fatty acids (BCFAs) concentration (μmol/g of feces) in the patients' fecal samples at different time points. The lines inside the rectangles indicate the medians and the whiskers the maximum and minimum values. The dots outside the rectangles are suspected outliers. Comparisons were made with the Wilcoxon signed-rank test to examine changes over time (*t* = 0, *t* = 1, and *t* = 2).

Regarding the quantification of the SCFAs in feces, we observed no significant differences over time in the main SCFAs acetic, butyric, and propionic acids in the studied infants. However, branched chain fatty acids (BCFAs), related to the microbial catabolism of proteins, slightly increased after the SOC (1 week), but significantly diminished (*p* = 0.008) after 1 month of ingestion of intact milk proteins with diet (Figure 3). When we segregated the infants into two groups depending on the LGG ingestion, a significant decrease (*p* = 0.036) in butyric levels was observed after 1 month of milk, and dairy consumption (between *t* = 0 and *t* = 2) only in the 8 infants who had not previously consumed the probiotic (Table 2).

**Table 2:** Concentrations of main SCFAs over time in the fecal samples of the study.

**SCFAs**	**Time 0 (before SOC)**		**Time 1 (1 week after)**		**Time 2 (1 month after)**	
	**Previous intake of the probiotic LGG**					
	**No (*n* = 8)**	**Yes (*n* = 4)**	**No (*n* = 8)**	**Yes (*n* = 4)**	**No (*n* = 8)**	**Yes (*n* = 4)**
Butyric	**18.12 (14.02–21.28)**	25.65 (16.62–38.19)	17.93 (12.64–20.81)	17.31 (16.69–22.36)	10.23 (8.55–15.04)*	38.17 (27.50–47.06)
Acetic	64.03 (50.35–84.76)	73.70 (54.78–91.09)	61.00 (50.80–86.02)	61.04 (59.50–71.17)	60.03 (42.41–73.75)	93.86 (83.21–106.21)
Propionic	15.74 (7.47–17.68)	18.75 (15.29–22.19)	12.88 (7.00–16.64)	20.34 (17.00–26.51)	9.08 (6.53–16.79)	20.84 (6.85–37.58)

Infants were segregated based on the consumption or not of the probiotic LGG during the restriction period before the SOC. Concentrations (μmol/g of feces) are expressed as medians and interquartile ranges (between brackets).

Wilcoxon test for paired samples were used to evaluate differences in concentrations across time.

Significant differences (p < 0.05) are indicated in bold with an asterisk for comparisons against t = 0.

*SCFAs, Short-chain fatty acids SOC; standardized oral challenge. LGG; Lactobacillus rhamnosus GG*.

Fecal content of indolic compounds which are produced by bacterial fermentation of proteins, such indole, skatole, and *p*-cresol, are reported in Table 3. For this latter compound a significant reduction (*p* = 0.05) was observed after 1 month of milk tolerance and regular dairy consumption (*t* = 2). The same tendency was observed for the concentrations of indole in feces, with a reduction over time although in this case the differences were not statistically significant. Skatole, a microbial degradation product from tryptophan, significantly increased in the studied infants 1 week after the SOCs. When the infants were divided according to the previous probiotic intake we observed that this increase was particularly notably in the group of infants that did not ingested the probiotic LGG (*n* = 8), showing a significant decrease at *t* = 2 (1 month after the challenge).

**Table 3:** Concentrations of indolic compounds over time in the fecal samples collected from the infants of the study.

**Indolic compound**	**Time 0**		**Time 1 (1 week after)**		**Time 2 (1 month after)**	
	**All studied infants (*****n*** **=** **12)**					
Indole	166.28 (41.58–489.56)		176.2 (34.86–467.34)		84.6 (21.98–344.78)	
Skatole	**0.84 (0.22–13.44)**		13.36 (0.24–36.26)*		0.82 (0.1–9.54)	
*p*-cresol	293.86 (27.08–1064.54)		**263.72 (68.24–1227.16)**		**51.24 (16.36–588.04)**^#^	
	**Previous intake of the probiotic LGG**					
	**No (*****n*** **=** **8)**	**Yes (*****n*** **=** **4)**	**No (*****n*** **=** **8)**	**Yes (*****n*** **=** **4)**	**No (*****n*** **=** **8)**	**Yes (*****n*** **=** **4)**
Indole	75.21 (22.86–376.17)	360.91 (220.03–489.57)	84.65 (19.77–411.49)	396.91 (195.51–666.24)	148.77 (21.98–344.79)	84.59 (62.06–248.09)
Skatole	0.30 (0.21–13.45)	1.88 (1.03–75.04)	**7.32 (0.25–22.71)**	28.37 (9.75–157.76)	**2.18 (0.12–9.53)**^**#**^	0.78 (0.08–150.47)
*p*-cresol	46.67 (13.54–871.95)	746.04 (545.71–10064.54)	93.63 (39.11–635.67)	1532.74 (282.91–2823.40)	51.25 (16.35–588.04)	139.94 (30.36–868.17)

Concentrations (μM) are expressed as median and interquartile ranges (between brackets).

Wilcoxon tests for paired samples were used to evaluate differences in concentrations across time.

Significant differences (p < 0.05) are indicated in bold for comparisons with time 0^*^, and time 1^#^.

*LGG; Lactobacillus rhamnosus GG*.

### Changes in Fecal Cytokines

**Figure 4:**
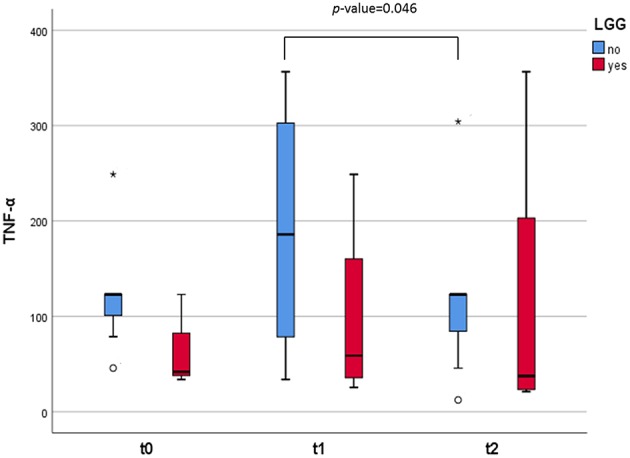
Box and whisker plots representing the concentrations (in pg/g) of TNF-α excreted in the fecal samples. The infants were divided in two groups: those who consumed the probiotic GG (*n* = 4, in red) and those who did not (*n* = 8, in blue). The lines inside the rectangles indicate the medians and the whiskers the maximum and minimum values. The dots outside the rectangles are suspected outliers (>1.5 × interquartile ranges) while the asterisks designate extreme value (>3 × interquartile ranges). Comparisons were made with the Wilcoxon signed-rank test to examine the differences over time (*t* = 0, *t* = 1, and *t* = 2).

The concentration of cytokines related to the Th1/Th2 balance was determined in the fecal water of the studied infants (Table 4). Among these, the concentration of IL-5 significantly increased 1 week after the SOCs (*p* = 0.04). On the other hand, the levels of gamma interferon (IFN-γ), an important cytokine related to inflammation, significantly decreased in the stool samples 1 month after the SOCs, and the regular introduction of CMP in the diet (*p* = 0.037). Additionally, we found differences in the levels of tumor necrosis factor (TNF-α) between infants who did not consume LGG and those who did (Figure 4). In particular, an increase in the levels of this cytokine was observed 1 week after the SOCs (*t* = 1) in the group of infants that did not consume the probiotic. The levels of this factor, crucial in inflammation, decreased significantly (*p* = 0.046) 1 month after the SOCs (*t* = 2), returning to the same levels observed before the SOCs. No significant differences over time in this immune factor were observed in the group of infants that previously consumed LGG. For the rest of the immune compounds determined in feces we did not observe any statistical difference between those infants who take (*n* = 4) or not (*n* = 8) LGG (Table S2).

**Table 4:** Concentration in pg/g of feces (expressed as median and IQR) of immune compounds in fecal samples collected in this study.

**Cytokines**	**Time 0 (before SOC)**	**Time 1 (1 week after)**	**Time 2 (1 month after)**
**Proinflamatory**			
IL-2	1.05 (1.05–1.05)	1.05 (1.05–1.42)	1.05 (1.05–1.05)
IL-12	104.49 (35.97–104.49)	104.49 (80.98–138.11)	75.77 (10.45–104.49)
IFN-ɤ	55.25 (47.75–173.17)	**149 (47.75**–**250.31)**	**47.75 (4.77**–**100.35)**^#^
**Anti-inflammatory**			
IL-4	0.15 (0.15–0.15)	0.15 (0.15–7.07)	0.15 (0.15–6.31)
IL-10	165.01 (29.85–237.02)	201.02 (29.85–298.54)	201.02 (29.85–276.18)
IL-13	1.72 (1.72–3.45)	2.59 (1.72–8.75)	2.59 (1.72–8.75)
IL-5	**4.75 (4.75**–**149.81)**	**61.71 (47.46**–101.89)*	49.91 (29.01–59.42)

Wilcoxon tests for paired samples were used to evaluate differences in concentrations across time.

Significant differences (p < 0.05) are indicated in bold for comparisons with time 0^*^, and time 1^#^.

*IQR, interquartile ranges; SOC, standardized oral challenge*.

## Discussion

In CMPA, CMP are excluded from the diet and infants are treated with formulas based on extensively hydrolyzed proteins or amino acids, which have been shown to be effective (24). These hypoallergenic substitutes and a dairy-free diet are currently the only therapeutic option available for the NIM-CMPA cases. However, the elimination of milk from the infant diet may lead to an increased risk of growth impairment (25), and dysbiosis with possible implications for future health (26). The impact of the absence of milk proteins and dairy products in the development of the infant's gut ecosystem remains poorly understood. Studies on gut microbiota in CMPA infants, particularly in NIM cases, have been scarce, and have only become available in recent years (10, 27, 28). In the present study, the gastrointestinal effect of SOCs in infants who outgrew their NIM-CMPA is reported. Also, this is the first time, as far as we know, that the introduction of intact milk proteins, and dairy products in the infant diet has been assessed in fecal samples, not only from a microbiological point of view, but also reporting changes in immune factors and metabolic products, and taking into account the possible effect of probiotic consumption during a hypoallergenic therapeutic period.

In our work we found that members of LAB, in particular the genus *Lactococcus*, significantly increased after the introduction of non-hydrolyzed CMP in infants who outgrew NIM-CMPA. This genus is commonly dominant in milk and fermented dairy products where it produces lactic acid from lactose. Hydrolyzed formulas normally used to treat NIM-CMPA infants are deprived of lactose, and its inclusion into the diet has been previously reported to modulate gut microbial composition by increasing LAB like lactobacilli (29). In agreement with this, the number of lactobacilli in feces increased significantly but only in those infants who did not consume LGG, most likely because in the infant' group ingesting the probiotic during the hypoallergenic diet before the SOCs, the levels of lactobacilli were already high at basal time (*t* = 0). This trend was observed initially by high-throughput sequencing of 16S rDNA amplicons and confirmed by qPCR assays targeting lactobacilli and other LAB (including members of the genera *Leuconostoc, Pediococcus*, and *Weissella*). Additionally, statistical differences in the increase of microbial diversity after regular introduction of conventional dairy products were observed in infants who were not consuming LGG. Infants whose diet was supplemented with the probiotic during the restriction period presented a higher fecal microbial richness.

On the other hand, we found significant decreases in some microbial groups belonging to the families *Lachnospiraceae*, and *Ruminococcaceae* after 1 month of the introduction of intact milk proteins, and normal dairy ingestion. A low relative abundance of *Ruminococcaceae* implicated in food sensitization has been described (30). In our study, members of this family, such as *Subdoligranulum* were significantly reduced after the introduction of whole CMP in the infants' diet. *Subdoligranulum*, a butyrate-producing member of the family *Ruminococcaceae*, has shown to be increased in children with food sensitization in early life (31). From the family *Lachnospiraceae*, the group of *E. hallii* has been suggested to be a key microbe with the potential to impact the intestinal metabolic balance. *E. hallii* participates in the formation of different SCFAs as butyrate and propionate (32). In our work, the reduction in sequences assigned to the group of *E. hallii* after 1 month of normal dairy ingestion was particularly significant in those infants that did not consume LGG, the same group of children in who we observed a reduction in fecal butyrate at this time point, which suggest a potential relation between both parameters. In fact, the concentrations of this anti-inflammatory metabolite was numerically higher in feces of the group that consumed the probiotic, in agreement with recent studies in NIM-CMPA that reported higher butyrate fecal levels when treated with LGG (10). The gut microbiota can determine to what extent dietary proteins are converted into other active metabolites, such as BCFAs, or different nitrogen containing compounds (33). Insufficient intake of food products, like milk and its derivatives, which are particularly important during infancy, can adversely affect the production of bacterial metabolites. SCFAs and tryptophan metabolites play a major role in the prevention of inflammatory diseases and are highlighted for their interaction with the immune system (34). In our study, we observed that fecal microbial metabolites derived from the fermentation of proteins, in particular BCFAs formed from branched-chain amino acids, diminished over time with the introduction of intact milk proteins into the diet of infants. It has been previously reported that the excretion of these BCFAs (isobutyric and isovaleric acids) in feces was higher in CMPA infants with milk restriction diets than in healthy ones with an unrestricted diet (12, 35). Other microbial metabolites from the breakdown of proteins, such as indolic compounds (derived from aromatic amino acids like tryptophan), were reduced after 1 month of regular incorporation of dairy into the diet. Milk proteins are particularly rich in tryptophan residues (36). Tryptophan is degraded to skatole and indole by microbial degradation in the intestine (23) with diverse gut microorganisms, such as lactobacilli, being able to catabolize this essential amino acid (34). In concordance, levels of skatole and indole, as well as lactobacilli, were higher at basal time in the group of infants consuming LGG, although the differences were not found statistically significant probably due to the small number of samples and patient's variability. Of note, among the indoles found in the fecal samples of the infants is *p*-cresol, derived from gut microbial degradation of dietary tyrosine (37), and which has been pointed as a pro-inflammatory, and pro-carcinogenic compound indicator of deprived intestinal health (38). Levels of this compound drastically diminished at the end of the period studied (non-restrictive diet) indicating a better digestion of proteins in the upper GIT and less colonic fermentation.

The precise mechanisms underlying in NIM-CMPA are almost unknown, presumably because this disease is difficult to diagnose, it is encountered mainly in human infancy and additionally, it is lack in experimental animals (39). In our study, although all infants had developed tolerance, we observed an increase in fecal levels of IFN-γ with the performance of the SOCs (1 week later). But the concentration of this Th1 cytokine significantly dropped after 1 month. IFN-γ plays an important role as an activator of antigen-presenting cells, and T cells, and deficiencies in IFN-γ response were described as being related to CMPA (40). Furthermore, we found differences over time in the levels of TNF-α excreted in feces of the two groups of infants. A fundamental role of TNF-α in NIM-CMPA has previously been indicated (41), as high concentrations of fecal TNF-α have been reported in children with inflammatory bowel disease and CMPA patients manifesting intestinal symptoms (42, 43). In a NIM-CMPA patient report case, Wada and colleagues reported the levels of fecal TNF-α to be increased for a long period after the oral milk challenge, suggesting that the induced inflammation results in long-term disturbance of the intestine (44). Our results also suggest an intestinal inflammatory response in the intestine of children subjected to oral milk challenges, despite tolerance acquisition. Nevertheless, we noticed these changes dropped to baseline levels after 1 month with increase dairy intake. Additionally, we observed that previous ingestion of the probiotic LGG may attenuate these responses to intact CMP improving gut homeostasis. This was in accordance with previous suggestions indicating that LGG can balance the production of cytokines possibly involved in CMPA (such as TNF-α and IFN-γ) (14), and lesser pro-inflammatory signaling (45). Although this is the first time, as far as we know, that these immune mediators are determined in fecal samples of NIM-CMPA infants.

## Conclusions

Although our study was only performed for a short period time and with a limited number of children, the results obtained showed for the first time that the introduction of milk, and dairy products in the infant's diet is followed by modifications in the infant's gut environment through changes in the microbiota (increases in lactic acid bacteria), and its protein metabolic fermentation end-products (decreases in branched chain fatty acids and *p*-cresol). Additionally, we corroborate that consumption by NIM-CMPA infants of the probiotic LGG during a hypoallergenic dairy-free diet with CMP hydrolysates may tune the microbial, metabolic, and immune profiles by driving beneficial downstream effects on the gut environment.

## Data Availability

The datasets generated for this study can be found in Sequence Read Archive (SRA) of the NCBI, SRR6884559, SRR6884560, SRR6884563, SRR6884564, SRR6884570 to SRR6884573, SRR6884575, and SRR6884577 to SRR6884579 for *t* = 0, and SRR7750516 to SRR7750539 for *t* = 1, and *t* = 2.

## Ethics Statement

The study was approved by the Regional Ethics Committee for Clinical Research of the Principado de Asturias (Ref. number 105/15). Personal data of the infants providing stool samples conformed to the ethical guidelines outlined in the Declaration of Helsinki and its amendments. Individual informed signed consent was obtained from all the families participating in the study.

## Author Contributions

SD and JD contributed with the conception of the study. LG and MD contributed equally to this work, performing both the laboratory determinations and statistics. JR provided the equipment and resources for the cytokine analysis. SJ, CM-N, DP-S, CB, and JD carried out the recruitment, diagnoses and follow up of the infants. JD was in charge of the SOCs and the recovery of information from the participant families. SD, MG, and AM planned the experimental design of the study and contributed to the interpretation of the data. SD provided material and human resources. JD drafted the manuscript. All authors corrected and approved the final version.

### Conflict of Interest Statement

The authors declare that the research was conducted in the absence of any commercial or financial relationships that could be construed as a potential conflict of interest.
