# Supplementary material for: Fecal Changes Following Introduction of Milk in Infants With Outgrowing Non-IgE Cow's Milk Protein Allergy Are Influenced by Previous Consumption of the Probiotic LGG
Source: Front Immunol. 2019 Aug 2;10:1819. doi: 10.3389/fimmu.2019.01819 (PMC6689952; doi:10.3389/fimmu.2019.01819)
Supplement: Supplementary file 1 [file Table_1.docx]

**Table S1.** Data and clinical characteristics of the non-IgE mediated CMPA infants recruited in this study.

| Infant code | Age (months)^a^ | Sex | Breastfeeding^b^ | Type of formula | LGG consumption^c^ |
| --- | --- | --- | --- | --- | --- |
| 1 | 20 | Male | no | EHF (Blemil) | no |
| 2 | 17 | Male | no | EHF (Nutriben) | no |
| 8 | 10 | Female | yes | EHF (Novalac) | no |
| 12 | 18 | Female | no | EHF(Novalac) | yes (Bivos®) |
| 15 | 12 | Male | yes | EHF(Nutramigen LGG) | yes |
| 17 | 24 | Male | yes | EHF(Nutriben) | no |
| 21 | 17 | Male | no | EHF(Nutramigen LGG) | yes |
| 22 | 22 | Female | yes | EHF(Nutramigen LGG) | yes |
| 32 | 14 | Male | yes | EHF(Alfaré) | no |
| 33 | 18 | Female | yes | AAF (Neocate) | no |
| 36 | 11 | Female | no | EHF (Althéra) | no |
| 41 | 25 | Female | yes | EHF (Almirón) | no |

^a^ At the time of sampling before the oral challenge.

^b^ Breastfeeding was considered if it was beyond first month of life.

^c^ Probiotic consumption was performed during the previous six months with hypoallergenic exclusion diet and before the oral milk challenge.

Abbreviations: CMPA, cow`s milk protein allergy; EHF, extensively hydrolyzed formula; AAF; amino acid-based formula; LGG; *Lactobacillus rhamnosus* GG
