# Supplementary material for: Fecal Changes Following Introduction of Milk in Infants With Outgrowing Non-IgE Cow's Milk Protein Allergy Are Influenced by Previous Consumption of the Probiotic LGG
Source: Front Immunol. 2019 Aug 2;10:1819. doi: 10.3389/fimmu.2019.01819 (PMC6689952; doi:10.3389/fimmu.2019.01819)
Supplement: Supplementary file 2 [file Table_2.docx]

**Table S2.** Concentration in pg/g of feces (expressed as median and IQR) of immune compounds in fecal samples collected in this study.

| Cytokines | | Time 0 (before SOC) | | Time 1 (one week after) | | Time 2 (one month after) | |
| --- | --- | --- | --- | --- | --- | --- | --- |
|  |  | Probiotic intake | | | | | |
|  |  | No (n=8) | Yes (n=4) | No (n=8) | Yes (n=4) | No (n=8) | Yes (n=4) |
| Proinflamatory | |  |  |  |  |  |  |
| IL-2 | 1.05 (1.05-1.42) | | 1.05 (1.05-1.05) | 1.05 (1.05-1.42) | 1.05 (1.05-1.42) | 1.05 (1.05-1.05) | 1.05 (1.05-9.40) |
| IL-12 | 104.49 (89.48-104.49) | | 57.47 (10.45-319.31) | 104.49 (104.49-138.11) | 57.47 (10.45-539.31) | 90.13 (59.44-104.49) | 10.45 (10.45-44.02) |
| IFN-ɣ | 47.75 (37.00-146.48) | | 134.98 (59.00-209.30) | 91.96 (47.75-174.72) | 268.32 (161.47-494.06) | 47.75 (4.77-93.64) | 83.33 (37.00-138.85) |
| Anti-inflammatory |  | |  |  |  |  |  |
| IL-4 | 0.15 (0.15-0.15) | | 0.15 (0.15-1.11) | 0.15 (0.15-1.69) | 4.75 (0.15-11.23) | 0.15 (0.15-0.15) | 6.31 (4.77-8.81) |
| IL-10 | 237.02 (131.22-237.02) | | 29.85 (29.85-63.64) | 267.78 (151.14-298.54) | 29.85 (29.85-97.02) | 237.02 (131.22-276.18) | 29.85 (29.85-110.88) |
| IL-13 | 3.45 (1.72-3.45) | | 1.72 (1.72-1.72) | 3.45 (1.72-8.75) | 1.72 (1.72-3.47) | 3.45 (1.72-8.75) | 1.72 (1.72-3.47) |
| IL-5 | 4.75 (4.75-48.28) | | 72.43 (4.75-265.04) | 52.29 (47.46-66.36) | 112.11 (69.94-368.23) | 54.74 (44.86-59.42) | 26.01 (4.75-67.38) |

Abbreviations: IQR, interquartile ranges, SOC, standardized oral challenge.
